# Analysis of Strain Distribution in Common Clinical Designs of Posterior Implant-Supported Fixed Partial Restorations: Comparison between Six Configurations

**DOI:** 10.3390/jfb15020047

**Published:** 2024-02-18

**Authors:** Oded Gelfan, Joseph Nissan, Asaf Shely, Gil Ben-Izhack, Liat Chaushu, Esi Sharon, Ari Glikman, Eran Zenziper, Ofir Rosner

**Affiliations:** 1 Department of Prosthetic Dentistry, Goldschleger School of Dental Medicine, Tel-Aviv University, Tel-Aviv 69978, Israel; dr.oded.g@gmail.com (O.G.); nissandr@gmail.com (J.N.); asafshely@gmail.com (A.S.); gil.ben.izhack@gmail.com (G.B.-I.); ariglikman@gmail.com (A.G.); drzenziper@gmail.com (E.Z.); 2 Department of Peridontology, Goldschleger School of Dental Medicine, Tel-Aviv University, Tel-Aviv 69978, Israel; liat.chaushu@gmail.com; 3 Department of Prosthodontics, Hadassah Medical Center, Hebrew University of Jerusalem, Jerusalem 91120, Israel; esisharon@hadassah.org.il

**Keywords:** implants, fixed restoration, strain gauge, stress concentration

## Abstract

The configuration of implant-supported prostheses is considered to influence the magnitude of stress concentrations, affecting their survival rate. The purpose of this study is to determine, through strain gauge measurements during load application, the dispersion and magnitude of strain concentrations in different implant-supported prosthesis designs. All designs matched those commonly used in posterior partially edentulous states. Three implants were inserted into an epoxy resin model (PLM-4B Vishay Measurements Group Inc., Raleigh, NC, USA), allowing for the delivery of three- and four-unit crowns in different cemented configurations. Loads were applied at vertical and oblique directions over the cast crowns in six different configurations representing various posterior partially edentulous restorations. The readings from the strain gauges adhered to the implant necks’ presented data on implant strain. Prostheses including cantilevers showed the highest strain among the three-unit prostheses within the prosthetic complex, and three single units showed the least (8133 µs vs. 201 µs, respectively). Angulated load application also had a role in amplifying the strains recorded, resulting in total strains of between 3.5 and 20 times higher than during vertical loading in all configurations. It can be concluded that the configuration of implant-fixed partial prosthesis changes the loads engaging the restoration, the implant, and, probably, the supporting bone.

## 1. Introduction

The use of dental implants has become a very widespread and acceptable treatment modality for the replacement of missing teeth. The professional literature has published high success rates for long follow-up periods (95% at 10-year follow-up) for partial denture restorations alongside a low rate of failures and complications [1]. Although low in rate, these failures and complications call for an analysis of the possible causes behind them [2,3,4].

Complications in implant treatment are divided into biological complications, such as a progressive loss of marginal supporting bone, with or without inflammatory processes, and infections, and technical complications, which are far more common, such as the loosening or rupture of connection screws, fractures of the implant body and prosthetic components, veneer chipping, and crown decementation. Both categories of complications are generally attributed to biomechanical factors [4,5]. These factors include occlusal forces applied over the implant-supported restoration during functional and parafunctional activities, the quality and level of the supporting bone, the design of the occlusal morphology, the opposing dentition, and forces applied over the restoration in horizontal offset (forces or loads engaging the restoration outside the long axis of the implant) [6].

Failure may occur when the equilibrium between the loads and the resistance of the prosthetic elements or the interface between the implant and the supporting tissues is impinged, and the loads overcome the capacity of the prosthetic complex or bone tissue to withstand the forces applied [4,7,8]. Leung et al. showed crestal bone resorption around an implant-supported fixed partial denture which was exposed to overload, and later identified some regeneration of the surrounding bone after the removal of the overload [9]. 

Skalak considers the geometry of dental prostheses as having a crucial influence on load distribution and, therefore, in the overall success of implant-supported prostheses. Using a mathematical model, load application over a cantilever partial prosthesis yielded stress twice as high upon the implant proximal to the unsupported unit compared to a prosthesis not including unsupported units, where the stress concentration was moderated [7]. 

A retrospective research study, which documented the distribution of implant fractures by type of prosthesis and the presence of parafunction, revealed that 83% of failed prostheses included mesial or distal cantilevers [8]. Another research study verified, through a three-dimensional finite element analysis, the dispersion of stresses within the bone tissue surrounding implants, supporting three-unit fixed partial dentures including two implant cantilever prostheses [10]. The results revealed that modifying the position and number of supporting abutments (engaged within their corresponding implants) influenced the stress concentrations. Contrary to other studies, this study loaded all prosthesis units simultaneously.

Kim et al.’s research used photo-elastic models and strain gauge analysis on several restorative configurations [11]. Their research also found high load concentrations around the distal implant of a three-unit cantilever prosthesis at the implant level and at the bone level. In order to reduce such potentially detrimental effects of such stresses, Takayama suggested that the cantilever should not extend beyond the distance between the implants to keep the mechanical advantage (also known as the force multiplier) less than one [12]. Such a design may turn a three-unit, two-implant cantilever prosthesis into a four-unit, two-implant cantilever prosthesis (with the addition of a central pontic).

Cemented implant-supported crowns are a very popular modality in the partially edentulous patient [13]. Their use is popular, frequent, safe, and comfortable [14,15,16]. Learning about actual stress and strain distribution among cemented restoration is viable through strain gauge analysis [17]. Strain registration provides the actual occurrence of strains arising within items and media under force application with no intermediate interface of a mathematical model such, as through finite element analysis [18].

Acka et al. also used strain gauge analysis on three-unit cemented restorations simulating an implant-supported bridge with a middle pontic. Central loads yielded significantly less strains than lateral loads [18]. Cehreli et al. used the same strain gauge setting, but with four-unit-implant cemented restorations, and reaffirmed significantly greater strains with offset loading applied over the prosthesis compared with a vertical load running parallel to the long axes of the implants [19].

The uniqueness of our experiment is that it aims to test common restorative configurations used to rehabilitate posterior partial edentulism under vertical and oblique (30-degree angle) occlusal loads and conditions. The experimental setting was designed to resemble oral conditions with simultaneous occlusal contacts on all restorative units, both vertical and oblique [20]. The findings of this study should shed light on potential technical failures, which are quite abundant, due to mechanical overload. Therefore, the purpose of this study is to analyze the magnitude and dispersion of load stresses in six various implant-supported cemented restorations in cases of posterior segmental edentulism.

The first working hypothesis is that the position and number of the supporting abutments will influence the loads transferred to the implants, manifested by the strains registered by the strain gauges. The second hypothesis is that applying loads at 30 degrees over the buccal cusps, although in the same magnitude, will change the dispersion of the loads and the magnitude of the strains displayed within the implants and the abutments attached to them. 

## 2. Materials and Methods

**Figure 1:**
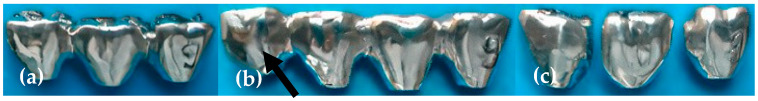
Photos of the cast crowns before cementation to abutments. (**a**) Three interconnected units, (**b**) 4 interconnected units with left cantilever (unsupported) unit (black arrow), and (**c**) 3 separated units.

**Figure 2:**
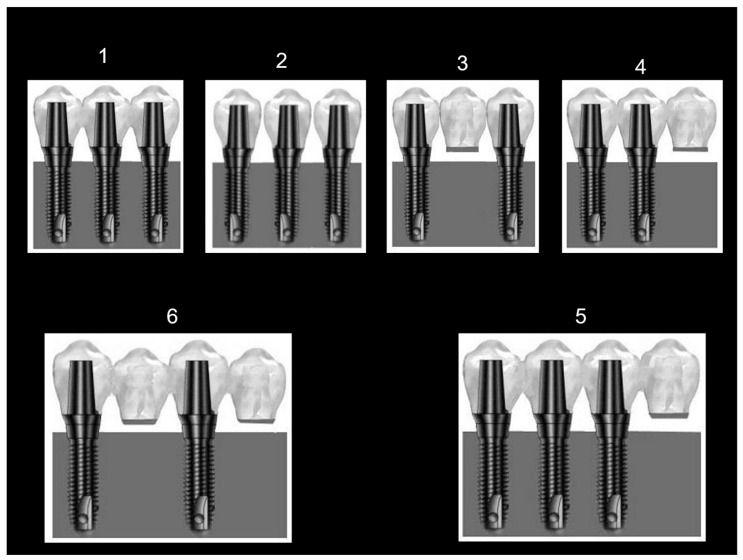
The 6 prosthetic configurations tested in our study. (**1**) Three interconnected units implant supported prostheses. (**2**) Three separated units. (**3**) Three-unit prosthesis with one central pontic. (**4**) Three-unit prosthesis with one cantilever. (**5**) Four-unit prosthesis with one cantilever. (**6**) Four-unit prosthesis with one pontic and one cantilever.

A resin block (PLM-4B Vishay Measurements Group Inc., Raleigh, NC, USA) was constructed, made of epoxy resin and characterized with a similar modulus of elasticity to human bone (2.8 GPa), with dimensions of 15.8 × 35.4 × 32 mm [21]. These dimensions ensure that the resin block will not crack during future drilling for implant installation. The length and width of the block are stock commercial dimensions, documented here in millimeters instead of inches. Three holes were drilled according to the standard surgical drilling protocol, with an increasing diameter at each step. Into the holes, three 3.8 mm diameter and 14 mm length dental implants were inserted (CP Grade IV Titanium; Density: 4.5 g/cm^3^, E: 104 GPa). The implant necks were 2 mm prominent over the model’s shoulders and 6 mm distant from each other. Transfer copings (CP Grade IV Titanium; Density: 4.5 g/cm^3^, E: 104 GPa) were screwed and inter-connected with acrylic resin (Dura lay, Reliance Dental Mfg Co., Worth, IL, USA) for stabilization and accuracy. An impression was taken by the open tray technique with a polyether-based material (Impregum, Espe, Germany) at the implant level with a custom-made acrylic resin tray. After the insertion of the analogues, a working cast was poured. Three cementation-type abutments (Sterioss cat #2235; Grade V Titanium: Ti-4Al-6V; Density: 4.4 g/cm^3^; E: 113 GPa) were fixed over the analogues at a controlled torque of 35 Ncm. Cast-base-metal cementable fixed prostheses were fabricated (Remanium CoCrMo alloy—GM380 by Dentaurum, Germany; composition: Cr: 29%; Mo: 4.5%; Co: 64.5%; Mn, C, Si: N2%; density: 8.2 g/cm^3^; E: 220 GPa) with an identical morphology (upper first molars) in six different configurations. The six configurations included four 3-unit prostheses and two 4-unit prostheses. The 3-unit prostheses were 3 connected crowns (config. No. 1); 3 separated crowns with interproximal contact points (config. No. 2); 3 connected crowns (bridge) with a middle pontic (unsupported unit, config. No. 3); and 3 connected crowns with a cantilever (unsupported unit) on one side (config. No. 4). The 4-unit prostheses included 4 connected crowns with a cantilever (unsupported unit) on one side (config. No. 5) and 4 connected crowns with a pontic interconnecting the first and third crowns and a cantilever as the fourth crown (config. No. 6). All configurations were cement-retained. These six configurations were chosen as they are popular among restorative dentists in the dental community and are encountered in treated patients of the department of oral rehabilitation in Tel Aviv University. The factors and considerations leading to choosing a certain restorative design depend on a patient’s economical constraints, inter-arch distance, availability of bone volume, and maintenance of a strict oral hygiene protocol. Examples of three cast restorations are depicted in Figure 1. After the fabrication of all six cast restorations, stock straight abutments were installed over the implants, each with the same torque of 35 Ncm, for each preplanned configuration (Figure 2). 

Two strain gauges (Strain-gauge EA-06-015EH-120, Vishay Measurement Group Inc. Raleigh N.C.; overall length: 0.64 mm; gauge length: 0.38 mm; overall width: 2.54 mm; grid width: 0.51 mm) were cemented with a designated glue (M-Bond 2000, Vishay Measurement Group Inc., Raleigh, NC, USA) horizontally onto the neck of each implant on the buccal and lingual aspects at a 180° opposite direction to each other. Horizontal mounting was possible within the space available and to facilitate the wiring of the gauge poles from the samples to the wiring adapter interface, described later.

During each loading session, the different restorations were cemented onto the abutments with temporary cement (Temp Bond NE, Kerr, CA, USA). The abutments were removed selectively when restorations including unsupported units (pontics/cantilevers) were tested. In configuration 2, the contact points of the individual restorations were fabricated so that dental floss passed with slight difficulty according to standard clinical procedure.

During load application over the restorations, readings from the strain gauges represented the electrical resistance changes. These changes occur as a result of the bending components (tension and compression) of the strain gauge caused by the deformation occurring at the implant neck. This deformation is a side effect resulting from the vertical and horizontal force fractions applied. The bending deformations at the implant neck necessitated their division into two types: tensile deformations, which were interpreted as positive calls, and compressive deformations, interpreted as negative calls. Our purpose was to determine the absolute strain values in order to sum them up and learn about potential fatigue damage to the implant necks.

The strain gauges were bonded with their designated glue (M-Bond 2000,Vishay Measurement Group Inc., Raleigh, NC, USA) to the implant’s neck. The strain gauges’ poles were connected to the wiring adapter interface through soldered leads (134 DFV, Vishay Measurement Group Inc., Raleigh, NC, USA) and thereafter to an electronic data scanner (Strain gauge Scanner: Model 5100 Instruments division, Vishay) and through a designated interface card mounted on a notebook PC docking station. 

**Figure 3:**
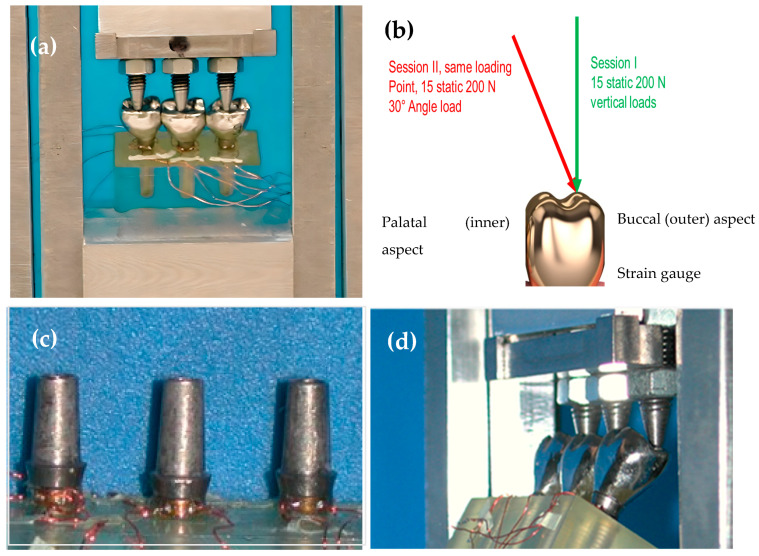
The loading apparatus. (**a**) A wide view of the load applicator. (**b**) A schematic of the vertical and oblique load applications. (**c**) A zoomed-in view with the strain gauges and abutments in place. (**d**) Load apparatus applying load on the restorations at a 30-degree angle.

A custom-made load applicator was fabricated (Figure 3). The appliance engaged 20 kg vertical (0°) and angled (30°) loads over each of the six restoration configurations through three or four individual pins to the inner inclines of the buccal cusps of each restoration, according to the tested configuration. A schematic drawing depicting the loading sessions on a single-unit level is illustrated in Figure 3b. Each loading pin was adjusted separately using a tightening screw to contact the opposite restorative unit (Figure 3d) with an 8 µ wide occluding paper until the paper was ruptured. Subsequently, the load applicator was calibrated to “zero”, from which ascending the load up to 200 N could begin. Each load was applied 15 times to each prosthesis to approximate the typical mean number of 15 chewing cycles that clinically prepare the food bolus with different consistencies for deglutition [22]. Each load lasted 10 s to allow for mechanical data acquisition, analysis by the computer, and relief. A 5 s rest session separated them to ensure the full recovery of elastic deformation between loads (up to 1 s) and to verify the resetting and rectification of the readings between sessions to a new zero value (up to 3 s). At the end of each session, after exchanging configurations, the calibration of the loading pins repeated itself.

For each loading session, strain gauge recordings were made. During extension or contraction, the strain gauge records changes in electrical resistance. The degree of distortion of the strain gauge is recorded in calculated micro-strain values, where

(1) $$Strain=\varepsilon =\frac{DL}{L}=\frac{DR}{R}$$

wherein DL represents the change in length of the strain gauge, L represents the initial length of the strain gauge, DR represents the change in electrical resistance in the strain gauge, and R represents the initial resistance of the strain gauge.

### Statistical Analysis 

The descriptive analysis consisted of the mean and standard deviation of the micro-strain values for each group. The groups were compared using a one-way parametric analysis of variance (ANOVA). *p* values of <0.05 were considered statistically significant. Specifically, an analysis was carried out through a series of variance analyses in order to verify the differences in stress distribution in the different prostheses’ configurations under vertical and angled load application.

Additionally, the differences between the results achieved from loading the different prosthesis configurations were compared through a Scheffe contrast analysis. An ANOVA was used to directly compare the difference in results between the vertical and angled load applications.

## 3. Results

The summary of the strain readings for vertical load is presented in Table 1, with statistical analyses in Table 2. The summary of the strain readings for 30° diagonal load is presented in Table 3, with statistical analyses in Table 4. 

Under vertical load conditions (Table 1 and Table 2), the results show significant statistical differences (*p* < 0.01) between the values read from all strain gauges in all six prosthesis configurations. The values interrelations differ at the implant level and at the strain gauge level (buccal vs. palatal). 

With regard to the three-unit prostheses, the lowest strain was recorded for the three separated units: 200.734 µs, which was distributed almost evenly among all three implants. Statistically (Scheffe contrast), the three-unit prosthesis configurations are located mostly in the groups with the lowest deformation levels.

The deformation values for the three-interconnected-unit configuration was 1376.534 µs, distributed over three implants. The deformation for this prosthesis was lower than the deformation value for the three-unit prosthesis with one central pontic (1879.1347 µs), which was distributed over two implants. Despite the differences in strain values between these two configurations, this was found to be statistically insignificant according to the Scheffe contrast. 

The highest strains were documented from loading the three-unit prostheses with one cantilever. This high reading of 7251 µs was recorded at the implant proximal to the cantilever. The total deformation value was 8133 µs, which was distributed between two implants. The deformation value for the implant distant from the cantilever was the lowest of all the values read in all configurations at 25.1 µs. Statistically (Scheffe contrast), the three-unit cantilever prosthesis was grouped with the configurations presenting the highest deformation values, due to the values read from the implant closest to the cantilever. 

Regarding the four-unit prostheses, the total deformation value for the four-unit prosthesis with one pontic and one cantilever, supported by two implants, was the lowest of all configurations tested (including all three-unit prostheses) at 141.0625 µs. Statistically (Scheffe contrast), this prosthesis is grouped with the low-deformation-value prostheses. 

The total deformation value for the four-unit prosthesis with one cantilever, supported by three implants, was 773 µs, which is a higher strain value compared with the previous four-unit prosthesis. Statistically (Scheffe contrast), this configuration disperses less load than the previous configuration.

The data from the 30-degree load application (Table 3 and Table 4) show significant differences (*p* < 0.01) between the strain values read from all gauges mounted on all implants in the six prosthesis configurations. The relationships between the strain values differ between the six prosthesis configurations based on the implant and at strain gauge locations (buccal or palatal). The overall deformation (strain) values were higher for the 30-degree load application compared with the values read under vertical load. The readings from the buccal-mounted strain gauges were significantly higher than those mounted on the palatal aspect, as expected, due to the load orientation.

Regarding the three-unit prostheses under the 30° load application, the three units with one cantilever prosthesis broke down under the 30-degree load application due to the rupture of the prosthetic screws. This configuration is considered the highest stress-concentrating configuration. The prosthesis with three units and one central pontic presented the lowest deformation among three-unit restorations under oblique load at 4579 µs, which was distributed on two implants. Statistically (Scheffe contrast), this prosthesis is grouped within the medium strain value configurations. The three-interconnected-unit prosthesis presented higher strain values compared with those read on the three-separated-unit prostheses (11133.2007 µs vs. 10876.136 µs, respectively), similar to when a vertical force was applied. The three-separated-unit configuration is statistically grouped with the low-strain-value prostheses.

Regarding the four-unit prostheses under the 30° load application, the following can be noted:

- On the implant level, the strain differences were low between the two configurations (1856.03 µs in configuration 6 compared to 1973.91 µs in configuration 5);
- These prostheses’ configurations did not show statistical significance (5921.75 µs vs. 3712.067 µs) (Scheffe contrast).

## 4. Discussion 

Long-term clinical trials that examine the success of partial implant-supported fixed partial dentures have reported high success rates, though some complications and detrimental factors were found [1,2,3]. Within the risk factors, the biomechanical factors are considered to provoke complications, including the fracture and dislodgement of different components of the restorations, the fracture of the implant fixture itself, and the early loss of peripheral bone support [5,6,8,13]. 

The maximal biting force measured in patients treated with implant-supported fixed partial dentures ranged between 200 N and 300 N for dentate and single-denture patients [23,24]. More recently, the mean occlusal bite force among dentate participants and partially edentulous patients restored with posterior implants was in the range of 236.1 ± 162.3 N to 275.7 ± 186.2 N [25]. Our study entailed 200 N loads in order to find out consequences during low-to-moderate force application. The clinical magnitude of the occluding forces applied on the restorations and the quality of the bone supporting the implants remain unmodifiable. Therefore, the clinician is left with partial control over the engagement and dispersion of loads, feasible through an adequate selection of prosthetic configuration and design [26,27].

Rangert et al. hypothesized that the linear geometry of fixed partial prostheses implies potential for creating tensile stress concentrations [8]. These forces are the non-axial fractions of the load applied over the prosthetic complex and initiate destructive stresses on it and the supporting bone. Akca et al., in contrast, compared, through a finite element analysis, the influence of linear placement versus staggered fixture placements (tripodisation) and found statistically significant differences in load dispersion between different prosthetic configurations [28]. Other publications showed different restoration characteristics which may influence the dispersion of the loads applied over the complex: the crown/implant ratio, cantilever length, occlusal table dimension, the distance between supporting implants, and the direction of the load. Forces defined as offset-loading are considered potentially detrimental and avoided where possible, because they result in bending forces with shear and tensile components [6].

Out of all potential loads applied over the prosthetic complex, the compressive force is best tolerated by the fixtures and the supporting bone. In a cantilever prosthesis, a type 1 lever is created and the implant proximal to the unsupported unit is exposed to high compressive loads while the adjacent implant is exposed to tensile loads. The length of the cantilever and the distance between the supporting implants are influential and are manifested by better strain distribution when the distance between the implants is greater, while load concentration is increased concomitantly with the cantilever length [29].

Occlusal loads, which include forces over the implants, are considered to be a major biomechanical factor and carry high importance for the long-term success of the restoration [2,4,7]. These loads result from the action of the occlusal forces applied over the restoration in function and parafunction [8]. The intraoral environment demonstrates mainly oblique occlusive forces [20]. These occlusal forces can be divided into different force fractions (vectors): vertical load fractions, which act closely parallel to the implant’s long axis and engage mostly compressive forces, and horizontal force fractions, which deviate and act in horizontal offset to the implant’s long axis, engaging tensile and compressive forces over the implant [30]. 

The forces engaging over the long axis of the implant are considered less detrimental to the supporting tissue and carry lower risks for damaging restoration components and implants [31]. In contrast, the horizontal force fractions engage offset loads (which act as bending forces and torques) and are considered detrimental and undesirable [30]. In two previous strain gauge analyses, it was reported that a vertical 1500 N load yielded the same strain at the implant neck as a 30° angle 50 N load. Both experiments recorded 600 μs at the implant necks [32,33].

Our study examined the load transfer and stress distribution in different popular fixed partial prosthesis configurations. The uniqueness of the current work is the attempt to imitate the diverse occlusal forces acting within the masticatory system by applying load on all units simultaneously and in two directions: vertical and angled.

The results affirm the basic assumptions of our study:

1. Significant differences exist in the stress distribution between the different prosthesis configurations (*p* < 0.01). Among the three-unit prostheses, the three-separated-unit configuration demonstrated the least strain compared with the other restorations (Scheffe contrast);
2. The 30-degree load application showed significantly higher strain compared with the vertical load application, and its distribution was also modified (*p* < 0.01).

Additionally, two interesting findings were revealed:

1. The four-unit prosthesis with one pontic and one cantilever presented the lowest load strains in this study, among all configurations and under both loading conditions (Scheffe contrast);
2. The three-unit prosthesis with one cantilever presented unfavorable strains under vertical load application and collapsed under angled load application due to a fracture of the abutment’s screws.

The strain distribution revealed for the three-separated-unit configuration are in accordance with Brunsky et al., where stresses transferred through a prosthesis in which all units are interconnected and supported by implants may be higher compared with the stresses transferred through three separated units [30]. Moreover, they claim that the magnitude of load transferred to the implants in a three-interconnected-unit restoration may turn out to be even higher than the original load applied over the prosthesis. This is because the configuration creates torques. This result is also presented in the mathematical model presented by Skalak [7]. It is also in accordance with more recent strain gauge research showing that three separated units delivered the least amount of stress under multiple simultaneous vertical loads compared to three interconnected crowns [34].

The cantilever three-unit prosthesis showed high readings at the implant closest to the cantilever. Stegaroiu et al. discovered similar findings of force distribution presented in a finite element analysis model, where a cantilever prosthesis yielded the highest stresses compared to a three-unit conventional bridge and three interconnected crowns [9].

Our and Stegaroiu’s results corroborate the clinical findings from Rangert et al.’s in vivo retrospective research, which showed that 83% of failed prostheses include cantilevers [8]. Kreissl’s prospective long-term follow-up also revealed that of all fixed prostheses among participants with partial edentulism, fixed restorations with cantilever extension exhibited the lowest success rate at 68.6% [35]. Furthermore, Zurdo et al. reported higher failure rates with cantilever bridges compared to conventional bridges, with implant fracture as the main cause for failures [36]. 

This study included angled load, which adds another potential force existing in the masticatory system. The three-unit cantilever bridge showed a catastrophic failure mode, and this finding supports Skalak’s mathematical assumption, which attributes the amplification of torques to the restoration’s design and geometry. The torque created through a 30° load can reach strain values that are double the magnitude of strains inflicted by the original occlusal load applied. Under these conditions, there is little surprise that even low to moderate occlusal loads of 200 N yielded such a catastrophic outcome as with the three-unit cantilever bridge. These findings show that prostheses with this configuration may experience high stresses beyond the physical-level tolerance of the restoration–implant–bone complex and promote early failures [5].

An increase in the strain recorded on the implant necks under lateral load was also reported by Akca et al. [28]. In their research, off-axial loading significantly increased the stresses and strains on implant necks. These results were measured in a simple three-unit temporarily cemented bridge with a central pontic, as was our third configuration. The second part of their research confirmed the same through a finite element analysis model. The same was true with Cehreli et al. when testing their four-unit cemented configuration [19]. 

Testing the three-unit bridge configuration with central pontic in our study revealed that the stresses were significantly moderated compared to the rest of the three-unit configurations under oblique loading. This is in contrast to the vertical loading, where the simple bridge registered the second highest strain readings of all three-unit prostheses. 

Regarding the four-unit prosthesis with one pontic and one cantilever, it registered the lowest strains under both oblique and straight loadings. These findings confirm Brunsky et al.’s [30] claim, which attributes more favorable potential torque dispersion when supported units are more distant from each other. It also corroborates Takayama, who suggested that the cantilever’s dimensions should not extend beyond the distance between the implants to keep the mechanical advantage less than one times this distance [12].

The results of the current experiment need to be supported by additional long term, in vivo research which will examine different partial implant-supported restorations. Spreading the strains and stresses outside the realm of implants and the prosthetic complexes they carry, through additional in vitro studies, may reveal various additional aspects explaining biomechanical overload’s role in peri-implant bone resorption. 

Our study has several limitations, including its in vitro design. The strain gauge analysis was documented only on the buccolingual plane and only at the points on which the strain gauges were attached. Anteroposterior strains were not recorded due to absence of strain gauges in our study in these areas. Encountering functional clinical situations with straight vertical forces such as the ones applied in our experiment is very scarce. The force applied was equal on all loading points, unlike the oral environment, where force magnitude increases posteriorly [37].

## 5. Conclusions

The prosthetic design has an impact on stress distribution over the supporting implants.

Under straight axial loads, three-unit prostheses with three separate units yield the least strain on the supporting implants. Yet, conditions where pure axial forces engage implant-supported crowns are quite rare, primarily due to constraints of the existing bone and the position of the opposing dentition. Under these clinical conditions, splinting the posterior crowns is a viable option. 

Oblique forces will create substantially more strain and deformities within the implants and probably within the surrounding bone.

Replacing a central supporting implant with a pontic in a three-unit bridge does not necessarily put additional stress on the two remaining implants. 

In cases of posterior partial edentulism, the use of cantilevered prostheses should be considered with caution.

In cases of four missing teeth in the posterior region, the use of four-unit prostheses with a central pontic and mesial cantilever should not be ruled out offhand. 

## Figures and Tables

**Table 1:** Mean, standard deviation, and sums of strain gauge readings of 15 cycles for vertically loaded configurations. Each implant showed strains in the buccal (Sgb) and opposite palatal (Sgp) locations.

	Implant 1		Implant 2		Implant 3		Sum for All (Loaded Implants)
Restoration Type	Sg B1	SgP1	SgB2	SgP2	SgB3	SgP3	
1. 3CU 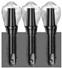	31.9333 ±6.6383	742.1333 ±52.4566	463.0667 ±132.3294	60.2 ±15.7852	54.2 ±5.2807	25.0007 ±11.9627	1376.534 (3 implants)
2. 3SU 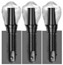	26 ±5.9161	14 ±3.5857	94.8667 ±11.4197	13.0007 ±4.779	28.5333 ±4.5019	24.3333 ±4.4186	200.734 (3 Implants)
3. 3UB 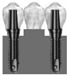	39.2 ±9.3595	1819.6667 ±243.0772			7.8013 ±5.2786	12.4667 ±3.7007	1879.1347 (2 Implants)
4. 3CUC 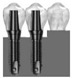	15.9375 ±11.891	9.125 ±4.0967	7251.625 ±1454.2769	857 ±60.3622			8133.6875 (2 implants)
5. 4CUC 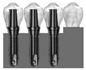	10.6 ±4.6721	286.4 ±8.4414	344.2 ±21.6768	24 ±4.6291	75.7333 ±4.667	32.6667 ±7.4322	773.6 (3 Implants)
6. 4UBC 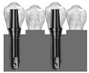	9.3125 ±6.9926	76.8125 ±18.9428			39 ±6.9857	15.9375 ±9.8757	141.0625 (2 implants)
1. 3CU = 3 connected units 2. 3SU = 3 single units 3. 3UB = 3-unit bridge (one pontic)				4. 3CUC = 3 connected units with one cantilever 5. 4CUC = 4 connected units with one cantilever 6. 4UBC = 4-unit bridge with one pontic and one cantilever			

**Table 2:** Variance (ANOVA) and Scheffe contrast analysis of the results read for the different prosthesis configurations under vertical load.

	Implant 1		Implant 2		Implant 3	
	SgB1	SgP1	SgB2	SgP2	SgB3	SgP3
ANOVA F(*p*) *	35.289 *	747.478 *	339.471*	2600.962 *	334.844 *	14.618 *
Scheffe Contrast	6, 5, 4 < 2, 1 < 3	4, 2, 6 < 5 < 1 < 3	2, 5, 1 < 4	2, 5 < 1 < 4	3 < 2 < 6 < 1 < 5	3, 6 < 2, 1 < 5

* *p* < 0.01.

**Table 3:** Mean, standard deviation, and sums of strain gauge readings of 15 cycles for obliquely (30°)-loaded configurations. Each implant showed strains in the buccal (Sgb) and opposite palatal (Sgp) locations.

	Implant 1		Implant 2		Implant 3		Sum for All Loaded Implants
Restoration Type	SgB1	SgP1	SgB2	SgP2	SgB3	SgP3	
1. 3CU 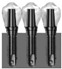	1150.5333 ±103.558	122.2 ±3.8582	7602.6667 ±488.4533	50.2673 ±21.1152	2100.2667 ±39.6835	107.2667 ±3.3481	11133.2007 (3 Implants)
2. 3SU 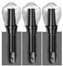	2465.1333 ±43.9641	24.1333 ±9.3034	5935.8667 ±110.8731	8.136 ±7.6302	2393.8667 ±124.5603	58 ±4.7809	10876.136 (3 Implants)
3. 3UB 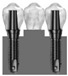	2161.3125 ±76.4231	115.75 ±4.1873			2095.9375 ±93.4783	206.375 ±5.6789	4579.375 (2 Implants)
4. 3CUC 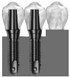	Abutment screws broke at load application						
5. 4CUC 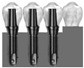	1775.375 ±26.7678	137.6875 ±4.1105	3280.1875 ±100.6321	329.3125 ±15.8228	214.125 ±47.5645	185.0625 ±6.7771	5921.75 (3 Implants)
6. 4UBC 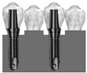	1720.1333 ±71.0723	170.1333 ±6.4461			1589.2667 ±50.0064	232.5333 ±5.9265	3712.067 (2 Implants)
1. 3CU = 3 connected units 2. 3SU = 3 single units 3. 3UB = 3-unit bridge (one pontic)			4. 3CUC = 3 connected units with one cantilever 5. 4CUC = 4 connected units with one cantilever 6. 4UBC = 4-unit bridge with one pontic and one cantilever				

**Table 4:** Variance (ANOVA) and Scheffe contrast analysis of the results read for the different prosthesis configurations under 30-degree buccal cusp load.

	Implant 1		Implant 2		Implant 3	
	SgB1	SgP1	SgB2	SgP2	Sgb3	SgP3
ANOVA F(*p*) *	774.695 *	1275.374 *	867.614 *	1895.265 *	1948.362 *	2698.658 *
Scheffe Contrast	1 < 6, 5, < 3 < 2	2 < 3, 1 < 5 < 6	5 < 2 < 1	2 < 1 < 5	5 < 6 < 3, 1 < 2	2 < 1 < 5 < 3 < 6

* *p* < 0.01.

## Data Availability

The data presented in this study are available on request from the corresponding author.

## References

[1] Hjalmarsson L., Gheisarifar M., Jemt T. (2016). A systematic review of survival of single implants as presented in longitudinal studies with a follow up of at least 10 years. Europ. J. Ora. Implantol..

[2] Chochlidakis k., Fraser D., Lampraki E., Einarsdottir E.R., Barmak A.B., Papaspyridakos P. (2020). Prosthesis Survival Rates and Prosthetic Complications of Implant-Supported Fixed Dental Prostheses in Partially Edentulous Patients. J. Prosthodont..

[3] Pjetursson B.E., Sailer I., Merino Higuera E., Spies B.C., Burkhardt F., Karasan D. (2023). Systematic review evaluating the influence of the prosthetic material and prosthetic design on the clinical outcomes of implant-supported multi-unit fixed dental prosthesis in the posterior area. Clin. Oral. Imp. Res..

[4] Eckert S.E., Salinas T.J., Akça K., Froum S.J. (2016). Implant fractures: Etiology, prevention and treatment. Dental Implant Complications: Etiology, Prevention and Treatment.

[5] Di Fiore A., Montagner M., Sivolella S., Stellini E., Yilmaz B., Brunello G. (2022). Peri-Implant Bone Loss and Overload: A Systematic Review Focusing on Occlusal Analysis through Digital and Analogic Methods. J. Clin. Med..

[6] Naert I., Duyck J., Vandamme K. (2012). Occlusal overload and bone/implant loss. Clin. Oral. Implant. Res..

[7] Skalak R. (1983). Biomechanical considerations in osseointegrated prostheses. J. Prosthet. Dent..

[8] Rangert B., Krogh H.J.P., Langer B., Van Roekel N. (1995). Bending Overload and Implant fracture: A retrospective Clinical Analysis. Int. J. Oral. Maxillofac. Implant..

[9] Leung K.C.M., Chow T.W., Wat Y.P., Comfort M.B. (2001). Peri-implant Bone Loss: Management of a Patient. Int. J. Oral. Maxillofac. Implant..

[10] Stegaroiu R., Takahiro S., Kusakari H. (1998). Influence of restoration Type on stress Distribution in Bone Around Implants: A three dimensional Finite Element Analysis. Int. J. Oral. Maxillofac. Implant..

[11] Kim W.D., Jacobson Z., Nathanson D. (1999). In vitro stress analyses of dental implants supporting screw-retained and cement-retained prostheses. Implant. Dent..

[12] Takayama H., Hobo S., Ichida E., Garcia C.T. (1989). Biomechanical considerations on osseointegrated implants. Osseointegration and Occlusal Rehabilitation.

[13] Lemos C.A.A., De Souza Batista V.E., Almeida D.A.D.F., Santiago Júnior J.F., Verri F.R., Pellizzer E.P. (2016). Evaluation of cement-retained versus screw-retained implant-supported restorations for marginal bone loss A systematic review and meta-analysis. J. Prosthet. Dent..

[14] Nissan j., Narobai D., Gross O., Ghelfan O., Chaushu G. (2011). Long-term outcome of cemented versus screw-retained implant-supported partial restorations. Int. J. Oral. Maxillofac. Implant..

[15] Altayyar S., Al-zordk W., Algabri R., Rajah E., Al-baadani A., Alqutaibi A.Y., Madina M.A., Ghazy M.H. (2023). Prospective evaluation of implants- supported, tooth-implant supported, and teeth-supported 3-unit posterior monolithic zirconia fixed restorations: Bite force and patient satisfaction. Clin. Exp. Dent. Res..

[16] Perea C., Del Río J., Preciado A., Lynch C.D., Celemín A., Castillo-Oyagüe R. (2015). Validation of the ‘Quality of Life with Implant Prostheses (QoLIP-10)’ questionnaire for wearers of cement-retained implant-supported restorations. J. Dent..

[17] Cehreli M. (2012). Biomechanics of dental implants. Handbook for Researces. Dental Science, Materials and Technology.

[18] Akca K., Cehreli M., Iplikcioglu H. (2002). A Comparison of Three-Dimensional Finite Element Stress Analysis with in Vitro Strain Gauge Measurements on Dental Implants. Int. J. Prosthodont..

[19] Cehreli M., Iplikcioglu H., Bilir O. (2002). The influence of the location of load transfer on strains around implants supporting four unit cement-retained fixed prostheses: In vitro evaluation of axial versus off-set loading. J. Oral. Rehab..

[20] Gibbs C.H., Lundeen H.C., Mahan P.E., Fujimoto J. (1981). Chewing movements in relation to border movements at the first molar. J. Prosthet. Dent..

[21] Vishay Precision Group Manual Bulletin #S–116H. https://micro-measurements.com/knowledge-base/instruction-bulletins.

[22] Nuvvula S., Sari K.I., Rafisa A. (2023). Chewing and swallowing patterns for different food textures in healthy subjects. Int. J. Dent..

[23] Morneburg T.R., Pröschel P.A. (2002). Measurement of masticatory forces and implant loads: A Methodologic Clinical Study. Int. J. Prosthodont..

[24] Mericske-Stern R., Zarb G.A. (1997). In vivo measurement of some functional aspects with mandibular fixed prostheses supported by implants. Clin. Oral. Implant. Res..

[25] Levartovsky S., Peleg G., Matalon S., Tsesis I., Rosen E. (2022). Maximal Bite Force Measured via Digital Bite Force Transducer in Subjects with or without Dental Implants—A Pilot Study. Appl. Sci..

[26] Rangert B.R., Sullivan R.M., Jemt T.M. (1997). Load factor control for implants in the posterior partially edentulous segment. Int. J. Oral. Maxillofac. Implant..

[27] Weinberg L.A. (1998). Reduction of implant loading using a modified Centric Occlusal anatomy. Int. J. Prosthodont..

[28] Akça K., Iplikçioglu H. (2001). Finite Element Stress Analysis of the Influence of Staggered Versus Straight Placement of Dental Implants. Int. J. Oral. Maxillofac. Implant..

[29] Misch C.E. (1990). Cantilever length and its relationship to biomechanical stress. Misch Implant Institute Manual.

[30] Brunski J.B., Puleo D.A., Nanci A. (2000). Biomaterials and biomechanics of oral and maxillofacial implants: Current status. Int. J. Oral. Maxillofac. Implant..

[31] Branemark P.I. (1983). Osseointegration and its experimental background. J. Prosthet. Dent..

[32] Yang T.C., Maeda Y. (2013). The biomechanical effect of platform switching on external- and internal-connection implants. Int. J. Oral Maxillofac. Implant..

[33] Castro C.G., Zancopé K., Veríssimo C. (2015). Strain analysis of different diameter Morse taper implants under overloading compressive conditions. Braz. Oral. Res..

[34] Nissan J., Ghelfan O., Gross M., Chaushu G. (2010). Analysis of load transfer and stress distribution by splinted and unsplinted implant-supported fixed cemented restorations. J. Oral. Rehab..

[35] Kreissl M.E., Gerds T., Muche R., Heydecke G., Strub J.R. (2007). Technical complications of implant-supported fixedpartial dentures in partially edentulous cases after anaverage observation period of 5 years. Clin. Oral Impl. Res..

[36] Zurdo J., Romão C., Wennström J.L. (2009). Survival and complication rates of implant-supported fixed partial dentures with cantilevers: A systematic review. Clin. Oral Implant. Res..

[37] Ferrario V.F., Sforza C., Serrao G., Dellavia C., Tartaglia G.M. (2004). Single tooth bite forces in healthy young adults. J. Oral. Rehabil..

